# Melt-Spun Photoluminescent Polymer Optical Fibers for Color-Tunable Textile Illumination

**DOI:** 10.3390/ma14071740

**Published:** 2021-04-01

**Authors:** Konrad Jakubowski, Manfred Heuberger, Rudolf Hufenus

**Affiliations:** Laboratory of Advanced Fibers, Empa, Swiss Federal Laboratories for Materials Science and Technology, Lerchenfeldstrasse 5, 9014 St. Gallen, Switzerland; konrad.jakubowski@empa.ch (K.J.); manfred.heuberger@empa.ch (M.H.)

**Keywords:** polymer optical fiber, light conversion, photoluminescence, bicomponent melt-spinning, textile design

## Abstract

The increasing interest in luminescent waveguides, applied as light concentrators, sensing elements, or decorative illuminating systems, is fostering efforts to further expand their functionality. Yarns and textiles based on a combination of distinct melt-spun polymer optical fibers (POFs), doped with individual luminescent dyes, can be beneficial for such applications since they enable easy tuning of the color of emitted light. Based on the energy transfer occurring between differently dyed filaments within a yarn or textile, the collective emission properties of such assemblies are adjustable over a wide range. The presented study demonstrates this effect using multicolor, meltspun, and photoluminescent POFs to measure their superimposed photoluminescent emission spectra. By varying the concentration of luminophores in yarn and fabric composition, the overall color of the resulting photoluminescent textiles can be tailored by the recapturing of light escaping from individual POFs. The ensuing color space is a mean to address the needs of specific applications, such as decorative elements and textile illumination by UV down-conversion.

## 1. Introduction

The concentration of light, captured from a large area, on a small emission or conversion area is the desired goal in applications such as solar energy harvesting or free-space optical communication signal enhancement. It can be achieved with conventional optics, like mirrors and lenses [1,2], but such approaches are limited by the etendue of the incoming light [3]. Photoluminescence (PL) can overcome this limitation due to re-emission of light at a new location. When an external light illuminates a PL active waveguide, i.e., a transparent waveguide doped with a fluorophore, emission occurs within the device, thus effectively increasing the etendue when delivering light to an attached receiver [4,5]. This type of device is known as a luminescent solar concentrator (LSC) and has been realized in the form of either planar or cylindrical waveguides [6,7,8,9]. Furthermore, PL waveguides are also utilized for optical communication, sensing, and illumination [10,11,12,13,14,15,16].

The PL waveguides studied in this work are polymer optical fibers (POFs) that are produced by melt-spinning which is the most commonly used method for manufacturing synthetic fibers [17]. Photoluminescent polymer optical fibers (PL-POFs) [18] have already been successfully utilized as LSCs for photovoltaic cell enhancement [10,19] and to amplify signal detection of free-space optical communication systems [20,21]. They consist of a high refractive index (RI) core, doped with a luminophore, where light conversion and re-emission occurs. It is advantageous to enclose the waveguiding core with a low-RI cladding which provides enhanced mechanical and optical stability [22].

Weaving is a well-established technique to translate fibers into textiles. It has been shown that POFs, woven into fabrics, can successfully be utilized as flexible devices for noninvasive body monitoring [23,24]. PL-POF-based textiles are known to exhibit improved light conversion as compared to single fibers, due to the reflection and shading effects caused by adjacent fibers [25]. In this work, we focus on the optical performance of yarns and textiles produced from PL-POF variants containing two different luminescent dyes (multicolor textiles). The subject of interest here is a nonresonant fiber-to-fiber light transfer that is different from a resonant energy transfer [26,27,28]. Fiber-to-fiber transfer is based purely on the re-adsorption of escaping light. In any PL waveguide, a certain portion of the fluorescent light escapes beyond the so-called trapping efficiency [29] which is a function of RI. In the multifiber yarn system proposed in this study, the light that escapes one fiber at a particular wavelength (color) enters adjacent fibers that contain a different dye that can reabsorb/convert the light, which gives rise to collective illumination effects.

PL-POF assemblies, doped with a combination of luminophores, might be an interesting strategy for optimization of light-harvesting, thanks to the extended absorption/conversion range [30,31]. However, employing differently colored fibers does not alleviate intrafiber self-absorption [32]; only part of the light that would otherwise be radiated away from the system is recaptured by adjacent waveguides. Adjusting the emission color of luminescent textiles by carefully selecting unicolor PL-POFs is crucial in such systems, and it is application specific, e.g., as decorative elements or colored emitters under white-light illumination [15,33,34,35,36]. The present study shows, to the best of our knowledge, for the first time, how the effect of fiber-to-fiber light transfer in multicolor PL textiles can be utilized and optimized towards color-tunability based on the two principles textile composition and dye concentration of the individual fibers constituting the fabrics.

## 2. Materials and Methods

### 2.1. Materials

**Figure 1:**
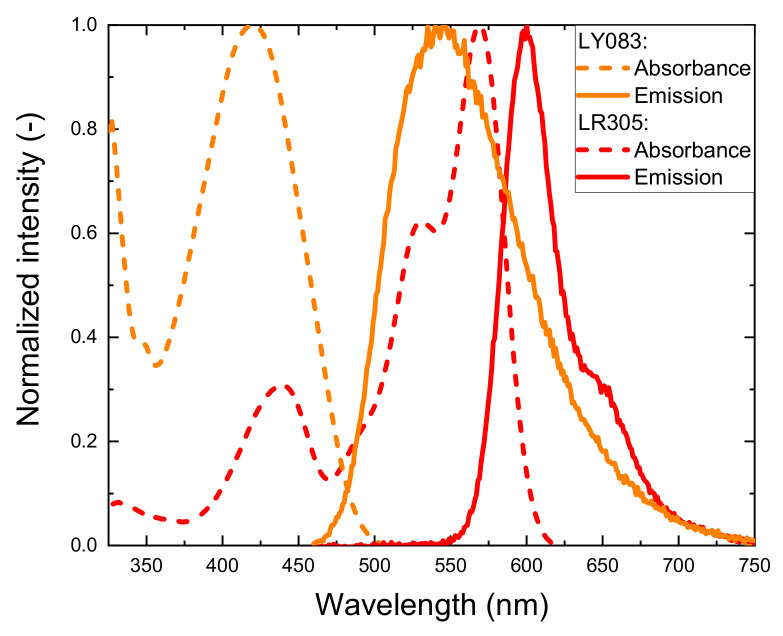
Absorption (dashed) and emission (solid line) spectra of LY083 (orange curve) and LR305 (red curve), measured in acetone.

Polycarbonate (PC) granulate (Sabic Lexan 103R, RI = 1.58) was purchased from Lenorplastics AG (Aesch, Switzerland); the terpolymer of tetrafluoroethylene, hexafluoropropylene, and vinylidene fluoride (THVP) granulate (THVP 2030GZ, RI = 1.35) was purchased from 3M Deutschland GmbH (Seefeld, Germany). Luminescent dyes, Lumogen Red LR305 and Lumogen Yellow LY083, were provided by BASF Schweiz AG (Basel, Switzerland). Their normalized absorption and emission spectra, measured for 0.001 wt.% solutions in acetone, are presented in Figure 1. All materials were used without further purification. For weaving of sample fabrics, a plain polyamide 6.6 (PA 6.6) monofilament with 100 µm diameter, provided by Monosuisse AG (Emmen, Switzerland), was used as warp yarn.

### 2.2. Preparation of Photoluminescent POFs

**Figure 2:**
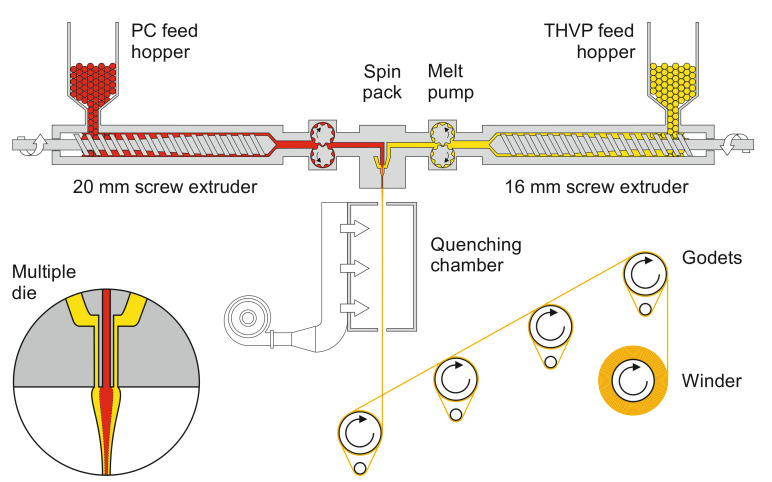
Schematic representation of the custom-made bicomponent melt-spinning plant used to prepare polymer optical fibers (POFs). Inset: multiple die, where the polymer melts meet just at the spinneret exit.

Polymer granulates were first dried for 12 h in a vacuum oven at 100 °C (PC) and 90 °C (THVP), respectively. LR305 or LY083 powder was then added to the dried PC granulates. To assure a uniform distribution of the dye, the granulate/powder mixtures were tumbled for 5 h. A bicomponent melt-spinning procedure was applied to prepare PL fibers with PC core and a semi-crystalline, low-RI THVP sheath (Figure 2).

The two polymers were fed from two separate single-screw extruders (Collin Lab & Pilot Solutions GmbH, Maitenbeth, Germany; 20 and 16 mm extruder for PC and THVP, respectively). The resulting polymer melt temperatures reached ~290–300 and ~270–280 °C for PC and THVP, respectively. Metering pumps (Mahr, Göttingen, Germany) with nominal throughputs between 3 and 9 cm^3^/min (core to sheath ratios between 45:55 and 50:50), transferred the melts into the spin pack. The resulting spin pressures reached between 74 and 128 bar. The fiber-forming spinneret comprised a multiple die [17,37] with a centered tube (0.8 mm inner and 1.2 mm outer diameter) within a 2.0 mm capillary. The bicomponent fiber exited the spinneret into a quenching chamber, where it was cooled by air in order to solidify before drawing. Finally, the fiber was taken up, drawn by four heated godets, and spooled onto a bobbin. The draw ratio, namely the ratio between the winding speed and that of the take-up godet, was varied within the range 2.0–2.2, resulting in fiber diameters of approx. 130 µm. Further process-related details are described elsewhere [19,37,38]. An overview of relevant information about the here-prepared fibers is summarized in Table 1. The designations, “F”, “U”, “R”, and “Y” stand for “fiber”, “undoped”, “red”, and “yellow”, respectively. Ultimate tensile strength and elongation at break of the undoped POF were determined as 13.3 ± 0.7 cN/tex and 57 ± 4%, respectively (measured on 20 samples with a StatimatME+, Textechno Herbert Stein, Mönchengladbach, Germany), indicating that the fiber’s toughness and flexibility allows for its textile processing.

### 2.3. Preparation of Photoluminescent Yarns

PC-based POFs, owing to their good mechanical properties, were used to prepare luminescent yarns, and to study the fiber-to-fiber energy transfer, which defines the yarns’ collective light emission performance. The yarns were prepared by manually twisting a predefined combination of PL-POFs, with the optional addition of a single undoped POF (FU_1) utilized as an extended light source. The yarn composition, i.e., the number of PL-POFs doped with 0.01 wt.% LR305 (FR_1) and of PL-POFs doped with 0.01 wt.% LY083 (FY_1) was varied to systematically quantify fiber-to-fiber light transfer. The actual combinations tested are further specified in the following sections.

### 2.4. Preparation of Photoluminescent Textiles

**Figure 3:**
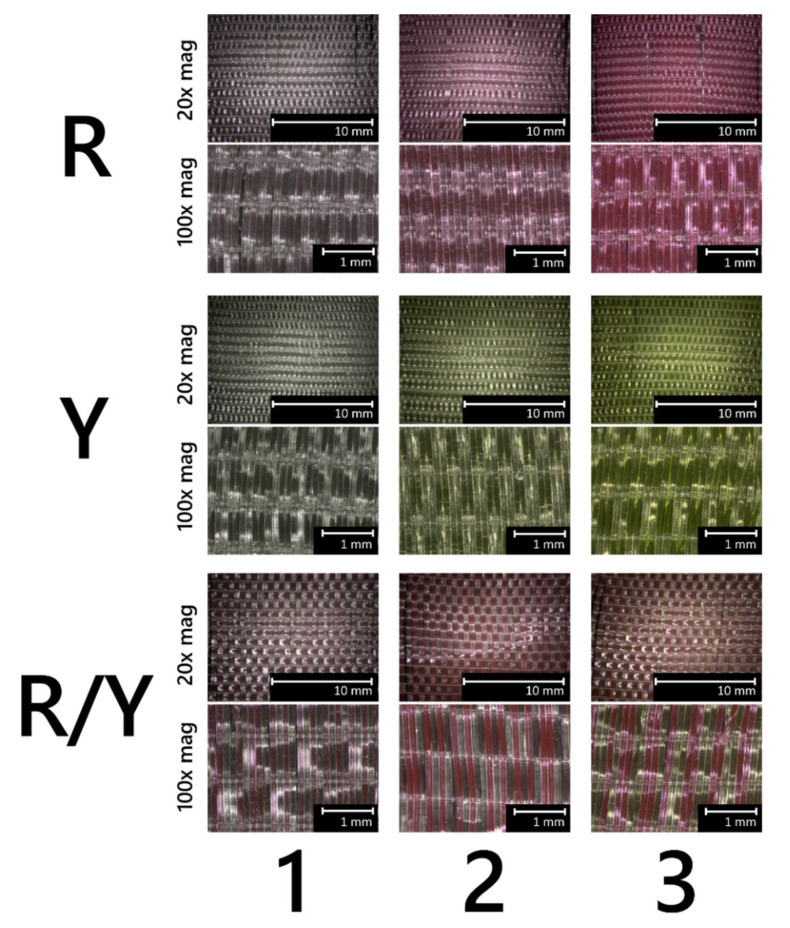
Microscopic images (reflected light) of here-prepared luminescent woven fabrics. Upper pictures were taken with 20x magnification, and bottom pictures with 100x magnification. “R”, “Y”, and “R/Y” stand for “red”, “yellow”, and “red/yellow”, respectively, and the numbers designate respective combinations as per Table 2.

To demonstrate the textile processing of the melt-spun bicomponent PL-POFs, prototype luminescent fabrics were woven on a customized narrow fabric loom (Jakob Müller AG, Frick, Switzerland). The loom comprises a weft needle which inserts a continuous yarn forward and backward across the narrow fabric during each shaft stroke, resulting in weft yarn pairs. The PL-POFs were applied as weft, across non-luminescent PA 6.6 monofilaments as warp. Overall, 14 different weft compositions were inweaved, as indicated in Table 2. Here, “R”, “Y”, and “R/Y” stand for “red”, “yellow”, and “red/yellow”, respectively, indicating whether the respective textile contained exclusively red fibers, yellow fibers, or a combination of these. In some cases, several filament pairs were inserted as weft at a time, as reported in Table 2. Figure 3 shows microscopic images of such prepared woven fabrics.

### 2.5. Fiber-to-Fiber Light Transfer Measurements

**Figure 4:**
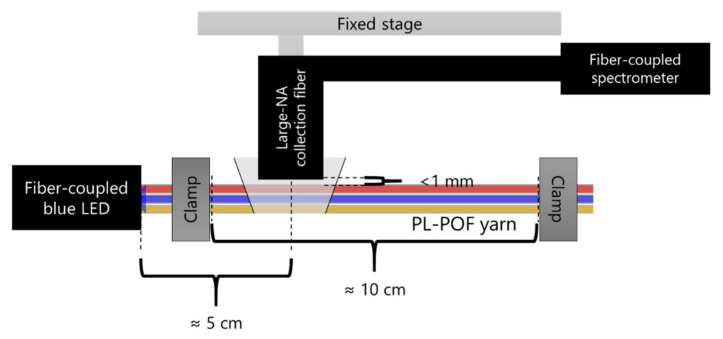
Schematic representation of the setup used to measure side-emitted light from PL-POF yarns. Illustrated is the case where the three fibers FU_1, FR_1 and FY_1 are combined (the twist is not shown).

To assess the energy transfer between PL-POFs within a hand-twisted yarn consisting of a combination of different fibers (FU_1, FR_1, FY_1), a measurement setup, as illustrated in Figure 4, was used. For illumination, an LED with an emission peak at 455 nm (M455F3, Thorlabs GmbH, Bergkirchen, Germany) was coupled via a glass optical fiber (M28L02, Thorlabs, Bergkirchen, Germany) and was illuminated the yarn end by utilizing a SubMiniature version A (SMA) to SMA mating sleeve (ADASMA, Thorlabs, Bergkirchen, Germany). The yarn end was optically terminated by a SMA905 multimode connector (B11050A, Thorlabs, Bergkirchen, Germany), attached to a universal bare fiber terminator clamp (BFT1, Thorlabs, Bergkirchen, Germany), and fixed straight between two clamps at a 10 cm distance. Side-emitted luminescent light was collected at a fixed longitudinal position utilizing a large numerical aperture glass fiber (M107L01, Thorlabs, Bergkirchen, Germany), directed orthogonally to the yarn’s axis, and led to a spectrometer (C10083MD, Hamamatsu Photonics, Solothurn, Switzerland).

**Figure 5:**
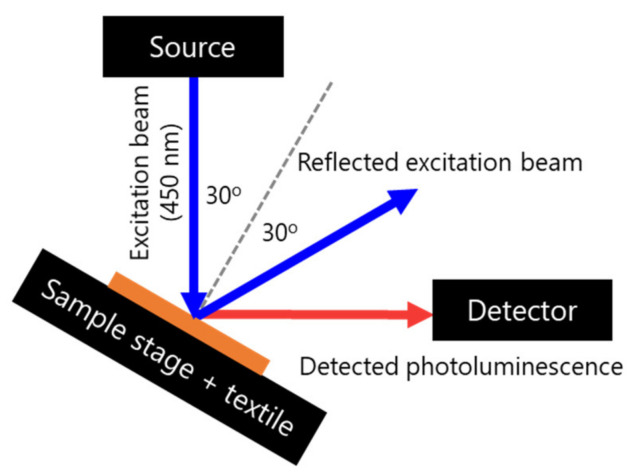
Front-face setup used to measure fluorescent emission from luminescent textiles. The tilt of the sample assured that the detection of the reflected excitation radiation was minimized.

Collective absorption and emission of the luminescent textiles was measured using a UV-Vis spectrophotometer (Cary 4000 UV-Vis, Agilent, Santa Clara, CA, USA). The spectra of the light emitted from the PL textiles were recorded using a spectrofluorometer (FluoroMax, Horiba Ltd., Kyoto, Japan) using a standard front-face method [39] with 450 nm excitation light, as illustrated in Figure 5. The sample was placed in a sample compartment preventing the external light from reaching the detector. Each measurement was repeated three times.

### 2.6. Measurements of Absorption and Emission Spectra of the Dyes in Acetone

The absorption and emission spectra of LR305 and LY083 were measured for 0.001 wt.% acetone solutions. The absorption spectrum was recorded using a spectrophotometer (Varian Cary 4000 UV-Vis Spectrophotometer, Agilent Technologies, Switzerland). The emission was recorded using a spectrofluorometer (FluoroMax, Horiba Ltd., Kyoto, Japan).

## 3. Results

### 3.1. Optical Performance of Photoluminescent Yarns

**Figure 6:**
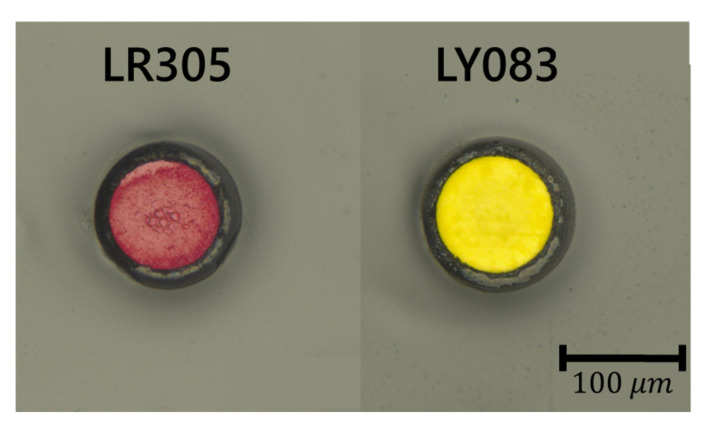
Microscopic pictures (in reflection) of PL-POFs with PC core and THVP cladding, doped with the photoluminescent dyes LR305 (fiber FR_1) and LY083 (fiber FY_1).

Figure 6 depicts cross-sectional images of the melt-spun PL-POFs FR_1 and FY_1. Respective core and fiber diameters were measured as 102 ± 2 and 134 ± 2 µm for FR_1, as well as 95 ± 3 and 126 ± 1 µm for FY_1, respectively.

**Figure 7:**
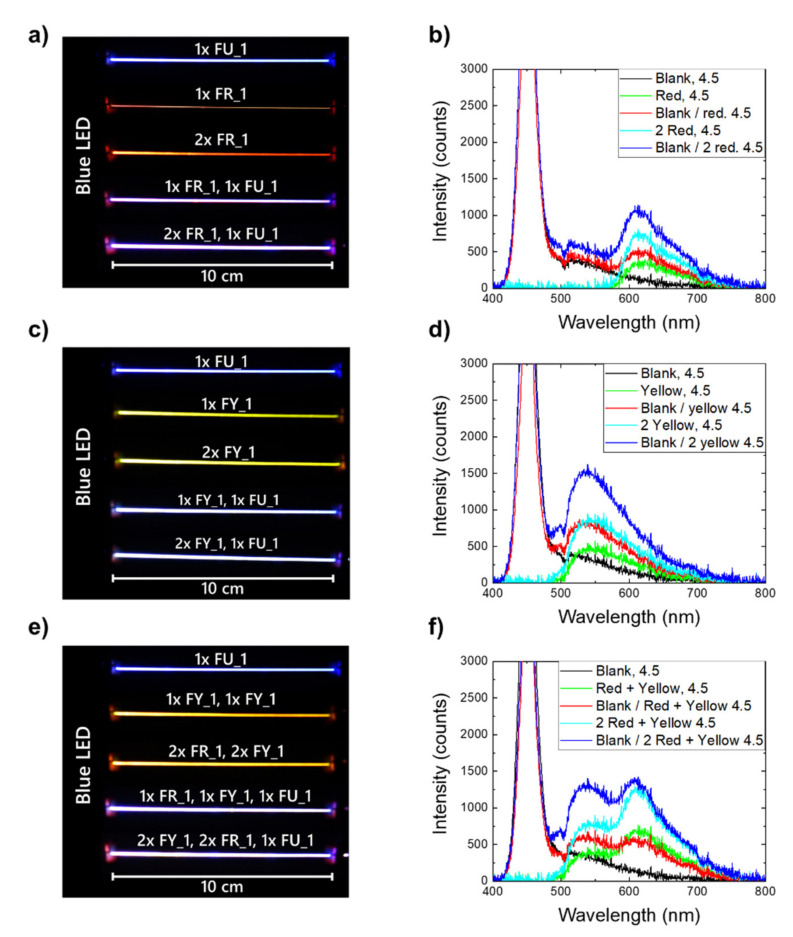
Photographs of yarns containing (**a**) red-doped and undoped POFs, (**c**) yellow-doped and undoped POFs, (**e**) red- and yellow-doped, as well as undoped POFs. In all cases, a blue LED is attached to the yarn’s left end. Images (**b**,**d**,**f**) show the corresponding emission spectra measured perpendicular to the yarns, 5 cm away from the LED-yarn connection.

The preparation and characterization of yarns produced from a combination of different POFs was performed as described in the Materials and Methods. Figure 7a shows yarns containing POFs with LR305-doped and/or undoped core with a blue (455 nm peak position) LED attached to one of their ends (i.e., to their left side in the picture). Figure 7b depicts the spectra those yarns emit laterally, measured at a distance of 5 cm from the illuminating diode. The yarn, consisting of only one single undoped POF (FU_1) laterally, emitted blue light due to scattering by the semicrystalline THPV sheath (cladding) [14]. While the light propagated inside the fiber, it underwent a total internal reflection at the core-cladding interface [40]. Due to the presence of RI inhomogeneity, a portion of this light was scattered out of the fiber [40] which served as excitation radiation for neighboring PL-POFs. The emission from PL-POF containing yarns exhibited a characteristic emission peak at around 600 nm, and the respective glow matched the color of the used dye (i.e., the blue LED light) and was converted into a luminescent light and outcoupled. Yarns, containing solely red PL-POFs, exhibited a strong, red-colored emission close to the LED which decayed over the photographed 10 cm length. When an undoped POF was combined with red fibers, the emission decayed less quickly and its color differed from that of doped-only fiber bundles. Increasing the number of red fibers in a yarn at constant illumination yielded higher intensity of the luminescent radiation since blue illumination was converted more efficiently.

Figure 7c displays the photographs of yarns containing yellow fibers, again under blue LED illumination from the left end. Figure 7d shows side-measured spectra of those yarns with a characteristic emission peak at around 550 nm. The emission behavior of the different yellow yarns was qualitatively similar, albeit, with a stronger luminescent peak signal which was due to the stronger absorption of blue light by LY083 dye (see Figure 1).

Another interesting method to tune collective color emission was to twist PL-POFs produced with two different dyes into a single yarn, thus, combining the luminescent emissions. Figure 7e shows the photographs of respectively mixed yarns with a 1:1 ratio of red and yellow fibers, while Figure 7f depicts their respective side-measured emission spectra.

Comparing the measured intensities of the characteristic peaks (approx. 550 and 600 nm for yellow and red fibers, respectively) of the different yarns (Figure 7b,d,f) suggested that the fibers with different dyes engaged in inter-fiber energy transfer. This effect was evident since combining red and yellow fibers resulted in higher intensities at around 600 nm (Figure 7f) compared to the case where only red-doped and undoped fibers constituted a yarn (Figure 7b). An analogous comparison with yellow-only yarns (Figure 7d) revealed that the intensities at around 550 nm decrease by similar amounts. Energy transfer between the fibers was possible since the emission of LY083 overlapped with the absorption of LR305 (Figure 1). This fiber-to-fiber energy transfer thus additionally affected the collective color balance of yarns comprising of distinct PL-POFs which is studied in more detail in the next section. Moreover, using separate fibers doped with distinct dyes, instead of combining several luminophores within one PL-POF, reduced the influence of self-absorption. This allowed for a longer propagation as well as distance of light, and, in consequence, for a more uniform color emission over yarn length. In addition, this prolonged distance may improve the efficiency of other devices based on PL-POFs such as LSCs [18,32,41]. However, such an approach would require a dedicated study aimed at understanding the potential offered by a fiber-to-fiber light transfer.

### 3.2. Optical Performance of Photoluminescent Textiles

**Figure 8:**
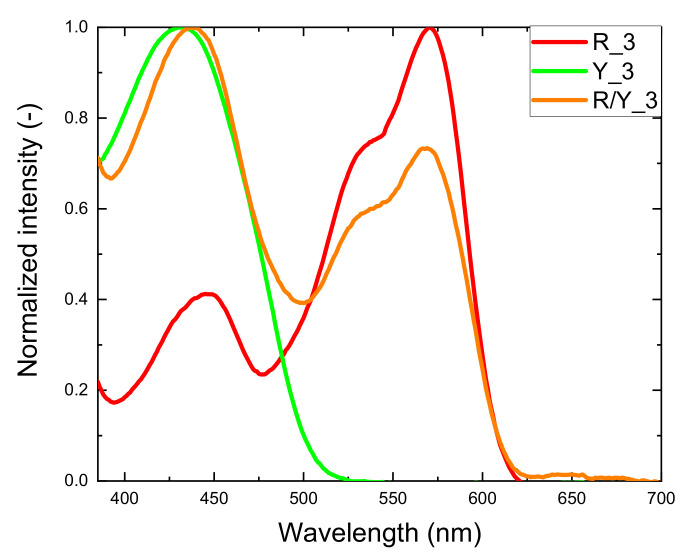
Examples of normalized absorbance spectra of three kinds of luminescent textiles: red fabric (R_3, red curve), yellow fabric (Y_3, green curve), fabric woven from a combination of PL-POFs (R/Y_3, orange curve).

A striking application example of PL-POF-based yarns are fashion textiles which are manufactured by weaving of assorted luminescent fibers into standard fabrics. The woven fabrics prepared in this study do not contain undoped POFs (Table 2); here, the external lamp acted as an excitation light source. The idea was that a spot illumination leads to a delocalized re-emission of converted light as a halo around the illumination spot. As shown in Figure 8, the light absorbance of a luminescent textile depends on the type of PL-POFs used in the woven fabric. The red curve (red fabric) directly reflects the absorbance of LR305 and the green (yellow fabric) that of LR083, while the absorbance of the textile produced from a 1:1 combination of red and yellow PL-POFs (orange curve) represents a combined spectra of both dyes (Figure 1).

**Figure 9:**
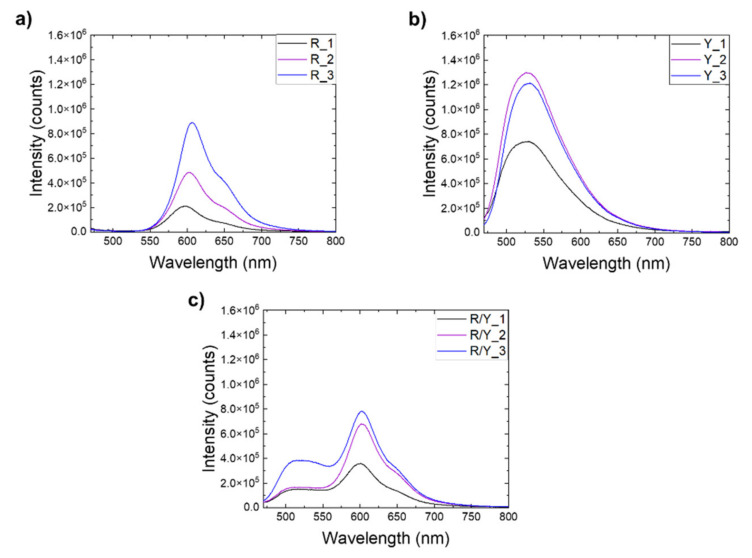
Emission spectra of (**a**) red textiles, (**b**) yellow textiles, and (**c**) two-colored textiles.

Figure 9 shows the emission spectra measured for different luminescent woven fabrics. As in the case of multicolored yarns (Figure 7), the emission from the textile fabric depends on the yarn composition.

**Figure 10:**
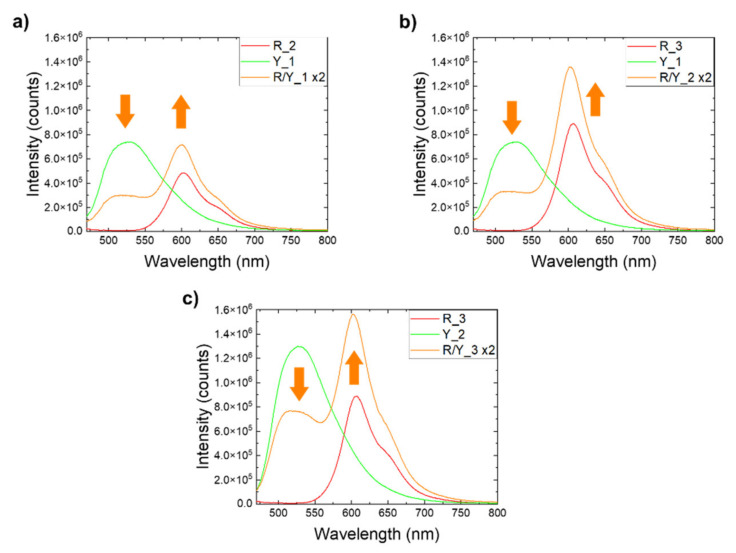
Emission spectra of luminescent textiles containing PL-POFs with both dyes (orange curve), compared to single-color fabrics containing the same PL-POFs (green and red curves). (**a**) Textiles made of fibers FR_2 and FY_1. (**b**) Textiles made of fibers FR_3 and FY_1. (**c**) Textiles made of fibers FR_3 and FY_2. Arrows indicate the energy transfer between PL-POFs.

To quantify the energy transfer between PL-POFs in the studied systems, selected variations of the respective fiber concentrations were used. The reference single-color textiles only contained PL-POFs of one color, while in the multicolor textiles, red and yellow fibers covered 50% of the total emitting area. In order to estimate the deviation of the emission spectrum of the multicolor textiles from a simple superposition of the single-color textiles’ emission spectra, we multiplied the intensity by the area factor (x2) for a quantitative comparison. Since the emission intensity was directly proportional to the emission area of the emitting species [42], this approach appeared valid as the used weft composition and the equal fiber diameters yielded a 1:1 emission area ratio. Figure 10 shows the emission spectra of the multicolor textiles adjusted in such a way which are compared to the emission spectra of single-color textiles prepared with the same PL-POFs as used in the respective multicolor systems.

As seen for the multicolor system in Figure 10, a deviation from the simple superposition of the spectra is observed for both characteristic emission peaks which is similar to the results observed for yarns (Figure 7). In every recorded case, the emission peak associated with LR305 (at around 600 nm) was stronger in two-color systems, while the emission peak associated with LY083 (at around 550 nm) became weaker. This is because the latter emission was absorbed by adjacent, red PL-POFs. The associated energy conversion and color change were associated with dissipation of part of the energy. This was expressed by a loss parameter  (Δ) obtained as $\Delta =1-\mathrm{AR}/Y/\left(AR+AY\right)$ for the respective samples, where AR/Y, AR, and AY were designated to the corresponding areas under the curves for multicolor, red and yellow samples. The sum $\left(AR+AY\right)$ was the expected superposition of the intensities. The calculated energy losses are shown in Table 3.

The largest energy loss was observed for the textile R/Y_1, where fibers with the lowest concentration of LR305 were used (FR_2). Thus, their ability to capture and convert light emitted by yellow fibers (FY_1) was limited. This was counteracted in the textile R/Y_2 by using red fibers with higher LR305 concentration (FR_3), which caused more light from yellow fibers to be recovered and converted. Increasing the concentration of LY083 in yellow fibers (FY_2) in R/Y_3 increased the amount of emitted yellow light available for conversion, however, the amount of LR305 in the red fibers required for conversion was not increased. The similarity of the deltas in R/Y_2 and R/Y_3 thus revealed a need for carefully balancing the dye concentration in multi-PL-POFs light converting textiles. Noteworthy, while increasing the concentration of the LR305 was beneficial to the performance of the device, an upper limit for this positive effect exists. It has been shown that increasing the dye concentration can lead to the formation of aggregates which are less efficient in emitting PL radiation [14,43,44]. This finding implied the existence of a maximum dye concentration at which the process ceases to be beneficial. Another study suggested that the optimum lies in the range between 0.05 wt.% and 0.1 wt.% for LR305 in PC [19].

**Figure 11:**
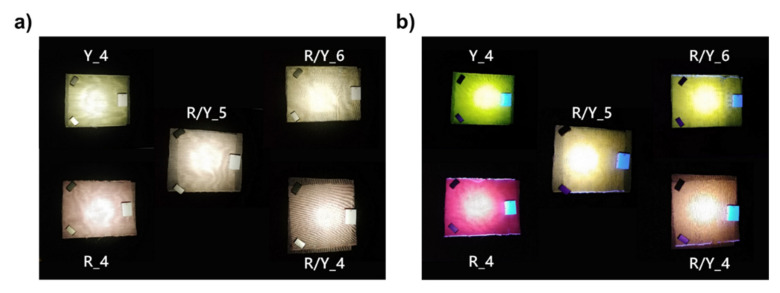
Luminescent textiles under (**a**) white light illumination and (**b**) UV light illumination. Captions indicate which fabrics were used, see Table 2.

Since PL dyes convert light to a lower energy, ultraviolet (UV) radiation can be used for illumination in down-conversion mode for visible light emission [45,46]. Consequently, the luminescent textiles developed in this study could also find applications as UV-conversion illumination fabrics. Thanks to the inherent waveguiding property of PL-POF fibers, a completely safe UV back-illumination scenario was straightforward. Figure 11 shows prototypes of PL textiles of different compositions (i.e., different content of red and yellow PL-POFs), exhibiting low-saturation colors when exposed to white light but vivid and bright colors when exposed to a 365-nm UV radiation in reflection mode.

## 4. Discussion and Conclusions

The paper demonstrates that interfiber light transfer and conversion occur within multicolor textiles. This result was obtained by mixing fibers containing different luminescent dyes and perform quantitative spectral analysis. Notably, it was found that the efficiency of the transfer increased significantly with an increasing concentration of the dye that was responsible for the conversion. This effect was demonstrated for two systems. First, luminescent yarns were prepared by twisting PL-POFs (which provide light emission and tunability of emitted color) together with a single undoped POF (which provides blue light required for photoluminescence). The second study was done on a textile luminescent system with an external lamp as excitation source utilizing a combination of PL-POFs as weft yarn in a woven fabric. Since light attenuation is of less concern when overhead illumination is considered, PC-based POFs are favored, due to their excellent ductility compared to, e.g., PMMA-based POFs [47].

This study shows how luminescent dyes utilized to dope-dye flexible POFs can broaden textile design possibilities impacting their color which, in turn, is an important design aspect vital to their commercial success. The demonstrators presented in this work show that it is possible to obtain aesthetically pleasant decorative elements by choosing differently colored luminescent dyes, varying their concentration in dope-dyed PL-POFs, and tuning the yarn’s composition with regard to the intended application. Note that it is easier to combine different prefabricated PL-POFs to achieve a desired luminescent color than to have to produce custom-designed fibers with a distinct combination of dyes. In addition to illumination and design, this approach could potentially also lead to a decrease in the inherent light leakage from fiber-based LSCs associated with trapping efficiency [29] or to textiles that can be used in anticounterfeit systems [15].

Another exciting implementation of luminescence are UV-down-conversion textiles for effective illumination in visible spectral region. Since respective fabrics are highly flexible and work without integrated electricity, they could find their ways into applications, such as lighting, tagging, or security markings. Using elaborate weaving technologies, PL-POFs could even be used to create two-phase, glow-in-the-dark, tessellated surface patterns with two distinctive design expressions under either daylight or UV illumination [48].

## Figures and Tables

**Table 1:** Overview of the prepared bicomponent PL-POFs.

Fiber Designation	Dye Concentration (wt.%)	Throughput Core/Sheath (cm^3^/min)	Melt Temperature Core/Sheath (°C)	Spin Pressure Core/Sheath (bar)	Draw Ratio	Winding Speed (m/min)
FU_1	None	7.5/9.0	298/280	94/68	2.0	900
FR_1	LR305 (0.01)	7.5/9.0	297/280	128/69	2.0	900
FY_1	LY083 (0.01)	7.5/9.0	298/280	110/69	2.2	1060
FR_2	LR305 (0.05)	3.0/3.0	291/269	93/117	2.1	410
FY_2	LY083 (0.05)	3.0/3.0	291/275	81/98	2.1	410
FR_3	LR305 (0.1)	3.0/3.0	291/269	81/98	2.1	410
FY_3	LY083 (0.1)	3.0/3.0	291/275	74/93	2.1	410

**Table 2:** Overview of the produced luminescent woven fabrics. Indicated are the type and respective number of PL-POFs inweaved per shaft stroke.

Fabric Designation	FR_1	FR_2	FR_3	FY_1	FY_2	FY_3
R_1	2					
R_2		2				
R_3			2			
R_4	8					
Y_1				2		
Y_2					2	
Y_3						2
Y_4				8		
R/Y_1		2		2		
R/Y_2			2	2		
R/Y_3			2		2	
R/Y_4	6			2		
R/Y_5	4			4		
R/Y_6	2			6		

**Table 3:** Integrated emission intensities of the graphs shown in Figure 10.

	R_2	R_3	Y_1	Y_2	R/Y_1	R/Y_2	R/Y_3
Integrated emission (counts × nm)	3.55 × 10^7^	6.30 × 10^7^	7.85 × 10^7^	1.32 × 10^7^	7.64 × 10^7^	1.23 × 10^8^	1.71 × 10^8^
Δ (-)	-	-	-	-	0.33	0.13	0.12

## Data Availability

Data sharing is not applicable to this article.
